# SIRT3: Striking at the heart of aging

**DOI:** 10.18632/aging.100256

**Published:** 2011-01-11

**Authors:** Yufei Liu, Dan Zhang, Danica Chen

**Affiliations:** ^1^Department of Molecular and Cell Biology, University of California, Berkeley, CA 94720, USA; ^2^Department of Nutritional Science and Toxicology, University of California, Berkeley, CA 94720, USA

Ever since the realization that aging is a regulated process, intense research has gone into uncovering the key players underlying its regulation. Among the most intriguing molecular factors that have been uncovered are the sirtuins, a family of NAD^+^-dependent deacetylases that have been implicated in lifespan extension in model organisms ranging from yeast to flies[1]. Mammals contain seven members of the sirtuin family, SIRT1-7, of which SIRT1 has been the most extensively characterized. Although the exact role of sirtuins in mammalian aging awaits further elucidation, a barrage of recent studies have shown that the mitochondrial sirtuin SIRT3 has an important role to play in warding off the vicissitudes of aging. In the previous issue of *Aging*, Hafner et al.(2010) add to the growing body of evidence by showing that mice lacking SIRT3 develop severe age-related pathologies in the heart [2].

Aging in the heart is characterized by hypertrophy and fibrosis, although its exact causes are unknown. Employing SIRT3−/− mice, Hafner et al. present intriguing evidence linking these aging phenotypes to mitochondrial dysfunction and SIRT3[2]. The authors show that the hearts of SIRT3−/− mice are more prone to aging phenotypes and are less resistant to stress. They also note that the hearts of SIRT3−/− mice contain mitochondria that are significantly more susceptible to swelling, a hallmark of aging. The mitochondrial permeability transition pore (mPTP), a multi-protein complex, is thought to be one of the main culprits driving age-related pathology in mitochondria[3]. Thus, inhibition of the mPTP could potentially have major therapeutic value. The authors found that SIRT3 interacts with and deacetylates cyclophilin D (CypD), a key component of the mPTP and a target of the prescription drug cyclosporine A (CsA) [4]. Interestingly, the lysine residue on CypD targeted by SIRT3 is adjacent to the CsA binding site, raising the possibility that deacetylation of CypD by SIRT3 might regulate its function. Whereas CsA also inhibits structurally-related cyclophilins, leading to suppression of the immune system, the findings of Hafner et al. open up the tantalizing possibility of targeting SIRT3 to specifically suppress the pernicious effects of CypD.

This study combined with recent work by Someya et al. (2010), Kim et al. (2010), and Sundaresan et al. (2009) show that SIRT3 can delay the onset of age-related pathologies in multiple tissues [2,5-7]. The present study also comes on the heels of studies by Qiu et al. (2010), Someya et al. (2010), and Tao et al. (2010) implicating SIRT3 in protection against oxidative stress, a major cause of age-related pathologies[6,8,9]. One of the targets uncovered, the mitochondrial antioxidant superoxide dismutase 2 (SOD2), has been linked to numerous longevity models. Thus, a strong case has been built for SIRT3 as a protector against the ravages of aging. However, whether SIRT3 can actually extend lifespan is still unknown. Also, while SIRT3 activity is thought to decrease with age due to the lower levels of NAD^+^, the aging phenotypes of SIRT3−/− mice are only apparent when the mice are older [2,10]. It is still a mystery as to why SIRT3−/− mice are phenotypically normal when they are young. Much work remains to be done to further understand the role that SIRT3 plays in the aging process, but the floodgates have been opened, and SIRT3 is emerging as an increasingly attractive target for small molecule activators to extend healthspan or even lifespan.
